# Longitudinal patterns of inflammatory mediators after acute HIV infection correlate to intact and total reservoir

**DOI:** 10.3389/fimmu.2023.1337316

**Published:** 2024-01-05

**Authors:** Jozefien De Clercq, Marie-Angélique De Scheerder, Virginie Mortier, Chris Verhofstede, Stefaan J. Vandecasteele, Sabine D. Allard, Coca Necsoi, Stéphane De Wit, Sarah Gerlo, Linos Vandekerckhove

**Affiliations:** ^1^ HIV Cure Research Center, Department of Internal Medicine and Pediatrics, Faculty of Medicine and Health Sciences, Ghent University, Ghent, Belgium; ^2^ Department of General Internal Medicine, Ghent University Hospital, Ghent, Belgium; ^3^ Department of Diagnostic Sciences, Aids Reference Laboratory, Ghent University, Ghent, Belgium; ^4^ Department of Nephrology and Infectious Diseases, AZ Sint-Jan, Bruges, Belgium; ^5^ Department of Internal Medicine, Universitair Ziekenhuis Brussel, Vrije Universiteit Brussel, Brussels, Belgium; ^6^ Department of Infectious Diseases, Saint-Pierre University Hospital, Université Libre de Bruxelles, Brussels, Belgium; ^7^ Department of Biomolecular Medicine, Faculty of Medicine and Health Sciences, Ghent University, Ghent, Belgium

**Keywords:** acute HIV infection, inflammation, plasma soluble mediators, cytokines, chemokines, early ART, HIV reservoir

## Abstract

**Background:**

Despite the beneficial effects of antiretroviral therapy (ART) initiation during acute HIV infection (AHI), residual immune activation remains a hallmark of treated HIV infection.

**Methods:**

Plasma concentrations of 40 mediators were measured longitudinally in 39 early treated participants of a Belgian AHI cohort (HIV+) and in 21 HIV-negative controls (HIV-). We investigated the association of the inflammatory profile with clinical presentation, plasma viral load, immunological parameters, and in-depth characterization of the HIV reservoir.

**Results:**

While levels of most soluble mediators normalized with suppressive ART, we demonstrated the persistence of a pro-inflammatory signature in early treated HIV+ participants in comparison to HIV- controls. Examination of these mediators demonstrated a correlation with their levels during AHI, which seemed to be viremia-driven, and suggested involvement of an activated myeloid compartment, IFN-γ-signaling, and inflammasome-related pathways. Interestingly, some of these pro-inflammatory mediators correlated with a larger reservoir size and slower reservoir decay. In contrast, we also identified soluble mediators which were associated with favorable effects on immunovirological outcomes and reservoir, both during and after AHI.

**Conclusion:**

These data highlight how the persistent pro-inflammatory profile observed in early ART treated individuals is shaped during AHI and is intertwined with viral dynamics

## 1 Introduction

Cytokines, chemokines, and growth factors are small intercellular signaling molecules which play pivotal roles in orchestrating innate and adaptive immune responses. They are produced by a variety of immune and non-immune cells and are crucial for the cross-talk between local and systemic immune processes. During acute HIV infection (AHI), the brief period between HIV acquisition and the full development of anti-HIV antibodies, these signaling molecules are first detected within the mucosal sites of infection, where they drive local inflammation. As HIV spreads systemically, a cytokine storm occurs, following a distinct pattern and timing. Rather than bulk immune activation, this phenomenon consists of sequential activation of different biological pathways, reflected by waves of cytokines and chemokines in plasma (1). This cytokine storm is accompanied by the characteristic clinical presentation of acute retroviral syndrome (ARS), a mononucleosis-like clinical image, which occurs shortly before peak viremia (2). Many soluble mediators are pleiotropic molecules and their role during the earliest events of HIV-infection is ambivalent. Pro-inflammatory chemokines are crucial in attracting both innate and adaptive effector cells to eliminate infected cells. However, these local inflammatory responses can also result in the depletion of specific cell populations or disruption of lymphoid tissues (3). Furthermore, the recruitment of CD4+ immune cells to sites of ongoing viral replication could potentially trigger new rounds of infection and the establishment of viral reservoirs. Common gamma chain cytokines can drive lymphocyte proliferation, thereby enhancing the development of HIV-specific adaptive immune responses, but might simultaneously increase the susceptibility of resting CD4 T cells for latent HIV infection (4). Other soluble mediators such as type I interferons and the HIV-suppressive chemokines CCL3 and CCL4 can have direct anti-viral effects, but are generated too late to prevent establishment of the viral reservoir (5). The complex network of soluble mediators during AHI can be viewed as a blueprint for the local and systemic immune responses generated during AHI. It has been postulated that differences in host immune responses impact the severity of ARS presentation and might contribute to long-term outcomes. Evidence from the pre-universal ART era shows a clear association between ARS presentation during AHI and faster disease progression (6–8). Similarly, the magnitude of the cytokine storm during acute infection negatively affects the clinical prognosis during chronic infection (9, 10).

By blocking HIV replication, antiretroviral therapy (ART) decreases plasma viral loads to undetectable levels. Early treatment for all people living with HIV (PLWH) is the new paradigm in HIV management, turning HIV-infection into a chronic condition (11). Nevertheless, suppressive ART does not fully abrogate the inflammatory signature seen in untreated HIV infection. Residual inflammation remains a hallmark of treated HIV infection and is a predictor for cardiovascular disease, cancer, and overall mortality (12–14). The pathophysiology behind this inflammatory state remains to be fully elucidated. Various factors have been proposed as potential contributors to chronic inflammation in PLWH, such as low-grade viral replication in anatomical sanctuary sites (15), sensing of defective viral RNA (16, 17), CMV co-infection (18), perturbations in the gut microbiome (19), and disruption of the gastro-intestinal barrier, leading to bacterial (20, 21) and fungal translocation (22, 23). The presence of low-grade inflammation has been observed even in PLWH who received ART during the earliest stages of HIV infection, suggesting that the imprint of the underlying causes occurs extremely early or persists despite viral suppression (24–28). Studying individuals diagnosed during AHI could provide an interesting window into the timing, extent, and mechanisms behind these inflammatory disruptions.

In this study, we characterized the inflammatory milieu in plasma in a Belgian multicentric AHI cohort by measuring 40 soluble mediators with a role in immune and inflammatory responses. Within this unique population of PLWH who started treatment very early, we identified specific soluble mediators that remained elevated during treatment. Moreover, we established links between the longitudinal inflammatory profiles and clinical presentation, immunological parameters, and a comprehensive understanding of the HIV reservoir.

## 2 Results

### 2.1 Cohort description

To investigate the impact of early ART initiation on residual inflammation in PLWH, we studied soluble mediators in longitudinal plasma samples from 39 very early treated HIV-positive (HIV+) participants selected from a Belgian multicentric AHI cohort and 21 HIV-negative participants (HIV-). In the HIV+ group the median age was 38.77, the large majority (92.31%) was male and the main risk group was MSM (**Table 1**). The median age in the HIV- participants was similar to the HIV+ group (36, p=0.74). However, the HIV- group was more gender-balanced (61.90% vs. 92.31% males, p=0.011) and consisted of a smaller proportion of gay/lesbian people compared to the HIV+ group (4.76% vs. 74.36%, p<0.001). HIV+ participants were stratified based on the Fiebig stage at diagnosis of HIV infection; 8 (20.51%) participants were diagnosed in Fiebig stage II, 7 (17.95%) in Fiebig stage III, 2 (5.13%) in Fiebig stage IV, 20 (51.28%) in Fiebig stage V, and 2 (5.13%) in early chronic infection, Fiebig stage VI.

**Table 1:** Characteristics of study participants.

Characteristic	Early treated HIV+	HIV- controls	P-value
	n=39	n=21	
Demographics			
**Age**	37 [30-44]	36 [32-51]	0.74
**Male**	36 [92.31]	13 [61.90]	**0.011**
**Ethnicity**			0.31
European	33 [84.62]	21 [100]	
African	2 [5.13]	0 [0.00]	
Asian	3 [7.69]	0 [0.00]	
Hispanic/Latino	1 [2.56]	0 [0.00]	
**Sexual orientation**			**<0.001**
Gay/lesbian	29 [74.36]	1 [4.76]	
Heterosexual	7 [17.95]	16 [76.19]	
Bi/pansexual	2 [5.13]	0 [0.00]	
Unknown	1 [2.56]	4 [19.05]	
Fiebig stage			
II	8 [20.51]	NA	
III	7 [17.95]	NA	
IV	2 [5.13]	NA	
V	20 [51.28]	NA	
VI	2 [5.13]	NA	
Subtype			
B	20 [51.28]	NA	
C	2 [5.13]	NA	
A	2 [5.13]	NA	
F1	4 [10.26]	NA	
CRF-AG	5 [12.82]	NA	
Undetermined	6 [15.38]	NA	
ART regimen initiated at diagnosis			
2nd generation INSTI + 2NRTI	30 [76.92]	NA	
2nd generation INSTI + 2NRTI+bPI	4 [10.26]	NA	
1st generation INSTI + 2NRTI +bPI	3 [7.69]	NA	
1st generation INSTI + 2NRTI	2 [5.13]	NA	

Data are represented as median [IQR] values or n[%]. Significant p-values are marked in bold. P-values were calculated with Chi square tests (categorical variables) and Mann Whitney U tests (continuous variables). ART, antiretroviral therapy; INSTI, integrase strand transfer inhibitor; NRTI, nucleoside reverse transcriptase inhibitor; bPI, boosted protease inhibitor; NA, not applicable.

**Figure 1:**
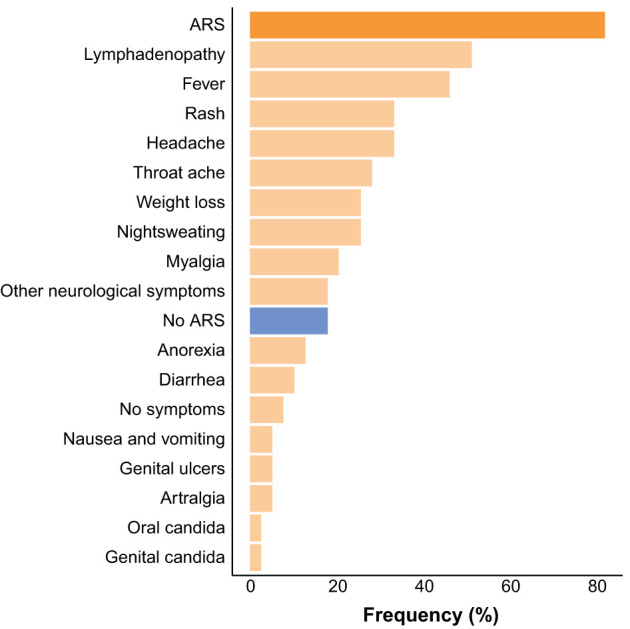
Frequency of acute retroviral syndrome (ARS) and ARS signs and symptoms at the time of HIV diagnosis.

Acute retroviral syndrome (ARS), defined as the presence of fever or ≥2 ARS symptoms at the time of diagnosis, was present in 32 (82.05%) of HIV+ participants. The most commonly reported signs and symptoms were lymphadenopathy, fever, and rash (**Figure 1**). On average, participants with ARS presented with 3 [IQR 2-5.25] signs or symptoms. In two participants fever was the only presenting symptom. There was a higher frequency of ARS in participants diagnosed in Fiebig stages II-III (14/15, 93.30%) versus Fiebig stages IV-VI (18/24, 75%), however this difference was not statistically significant (p=0.24). All HIV+ participants were placed on ART within 1 week after their first visit to the department.

The HIV+ group was followed longitudinally and plasma soluble mediators were measured on four timepoints: T0, during acute HIV infection; DVL at decreasing viral load on ART (median 157 copies/mL [IQR 74-322]) about one month (median 35 days [IQR 28-43]) after ART initiation; UD, after suppression of plasma viral load (median 278 days [IQR 176-365] after ART initiation); and UD+1, approximately one year later (median 726 days [IQR 623-817] after ART initiation).

### 2.2 Early ART initiation induces rapid viral suppression and restoration of peripheral immune blood cell counts

Acute HIV infection was hallmarked by a drop in absolute CD4 count, decreased CD4/CD8 ratio, decreased absolute B cell counts and high plasma viral load (**Table 2**). Participants who presented in Fiebig stages II-III had lower absolute CD4 count (p=0.0022) and higher plasma HIV viral load (p<0.001), compared to participants who presented in Fiebig stages IV-VI (**Supplementary Figures 1A, B**). On the other hand, participants in the later Fiebig stages had higher absolute CD8 counts (p<0.001) and lower CD4/CD8 ratios (p=0.036) (**Supplementary Figures 1C, D**). In contrast to previous studies (26, 29), HIV viral loads did not significantly differ between participants with and without ARS presentation (p=0.67) (**Supplementary Figure 1E**). However, there was a non-significant trend for lower CD4 T cell counts (p=0.11) and significantly higher levels of aspartate aminotransferase (AST) levels (p=0.028) in participants with ARS, the latter reflecting the systemic nature of ARS (**Supplementary Figures 1F, G**).

**Table 2:** Longitudinal laboratory parameters and HIV reservoir measurements in HIV+ participants.

Value	T0	DVL	UD	UD+1
**CD4 T cells (/µL)**	428 [326-595]	712 [566-979]**	818.05 [621.75-930.75]***	746 [602.5-931]***
**CD8 T cells (/µL)**	774.9 [321-1434.9]	686 [484-1055.9]	627 [502.38-867.25]	581 [473-730.5]
**CD4/CD8 ratio**	0.52 [0.36-0.86]	1.02 [0.77-1.46]*	1.07 [0.8-1.57]***	1.25 [0.98-1.8]***
**B cells (/µL)**	84.6 [62-125]	201 [144-226]*	206 [174-284]***	184 [165-244]***
**NK cells (/µL)**	186 [146-263.7]	246 [175-351.1]	235 [145.8-358.5]	248 [172-361.5]
**Monocytes (10^3^/µL)**	0.44 [0.35-0.61]	0.52 [0.44-0.71]	0.51 [0.43-0.63]	0.53 [0.46-0.6]
**Neutrophils (10^3^/µL)**	2.57 [1.92-3.37]	3.23 [2.09-4.08]	3.2 [2.19-3.96]	3.13 [2.3-4.09]
**Thrombocytes (10^3^/µL)**	198 [163-262.5]	238 [221-273.75]	235 [208.5-281]	235 [217.25-287]
**AST (U/L)**	30 [22.5-49.5]	22 [20-27]*	22 [18-25]	22.5 [20.25-25.75]*
**Plasma viral load (log10 copies/mL)**	6.51 [5.67-7]	2.2 [1.87-2.43]***	<50***	<50***
**Total HIV DNA (copies/10^6^ CD4 T cells)**	16574.77 [5095.11-47742.3]	NA	310.05 [148.61-679.54]***	114.25 [50.56-212.62]***
**Intact HIV DNA (copies/10^6^ CD4 T cells)**	6039.27 [2883.2-11662.87]	NA	212.29 [66.33-473.6]*	36.05 [14.81-82.64]***

Data are represented as median [IQR] values. Values which differed significantly from the measurements at the T0 timepoint are indicated with *, p<0.05; **, p<0.01; ***, p<0.001 and marked in bold. P-values were calculated with Friedman’s test and post-hoc Dunn’s test. AST, aspartate aminotransferase; NA, not applicable.

Two decades ago, the beneficial impact of initiating ART very early on immune recovery was well-established (30–32). In our early treated population, the median CD4/CD8 ratio normalized to >1, and the median CD4 count increased to 712/µL as soon as 1 month after treatment initiation (DVL). Additionally, normalization of the B cell subset was demonstrated at the DVL timepoint (**Table 2**). The median time to viral suppression was 97 days [IQR 90-131]. Neither Fiebig stage (Fiebig II-III: median 97 [IQR 92-152] days, Fiebig IV-VI: median 99 [IQR 89.5-130] days, p=0.98), nor clinical presentation (ARS: median 99.5 [IQR 94-154] days, no ARS: median 88 [IQR 71.5-99.5] days, p =0.096) at diagnosis had a significant impact on the time to viral suppression.

### 2.3 The intact HIV reservoir declines faster than the total HIV reservoir after early treated acute HIV infection

**Figure 2:**
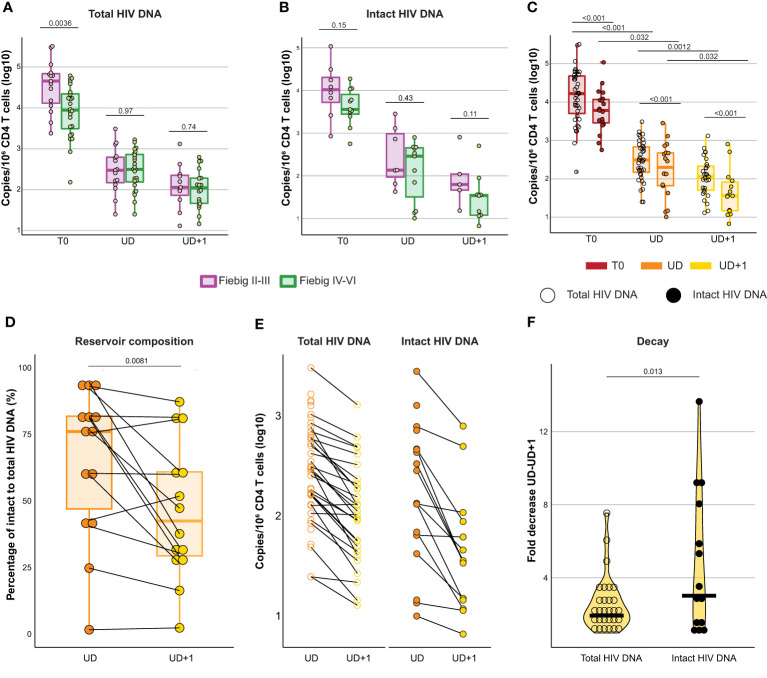
The intact HIV reservoir declines faster than the total HIV reservoir after early treated acute HIV infection. **(A, B)** Box plots displaying total **(A)** and intact **(B)** HIV DNA measurements (log10 transformed) at T0, UD, and UD+1 for HIV+ participants diagnosed and treated in Fiebig stages II-III (purple) and Fiebig stages IV-VI (green). Dots are colored according to Fiebig stages. P-values were calculated using Mann Whitney U tests. **(C)** Box plots displaying total (empty circles) and intact (filled circles) HIV DNA measurements (log10 transformed) at T0, UD, and UD+1. P-values comparing measurements across timepoints were calculated with Friedman's test and post-hoc Dunn's test. P-values comparing total and intact HIV DNA were calculated using paired Wilcoxon signed-rank tests. **(D)** Box plot displaying the ratio of intact to total HIV DNA (%) at UD (orange) and UD+1 (yellow). P-value was calculated using a paired Wilcoxon signed-rank test. **(E)**, Dot plot displaying the decline in HIV reservoir between UD (orange) and UD+1 (yellow) in longitudinally paired samples for total (empty circles) and intact (filled circles) HIV DNA. **(F)** Violin plot displaying the difference in fold decrease of the HIV reservoir between UD and UD+1 for total (empty circles) and intact (filled circles) HIV DNA. P-value was calculated using a paired Wilcoxon signed-rank test.

Treatment initiation during acute HIV infection curtails reservoir seeding and thereby limits the size of the reservoir (33–36). Total and intact viral reservoir was measured at T0, UD, and UD+1. In our cohort, total HIV DNA was significantly higher in Fiebig II-III compared to Fiebig stages IV-VI at T0 (p=0.0036, median 45231 and 8834 copies/10^6^ CD4 T cells respectively) (**Figure 2A**). However, no difference was found during suppressed viremia (UD: p=0.97, median 299 and 313 copies/10^6^ CD4 T cells and UD+1: p=0.74, median 114 and 133 copies/10^6^ CD4 T cells respectively). Even though total HIV DNA is a robust and subtype-independent measurement of the total cell-associated HIV load (37), it cannot distinguish between defective and intact proviruses. To distinguish the total from the intact reservoir, we performed a digital polymerase chain reaction (PCR)-based assay targeting the packaging signal (*psi*) and envelope (*env)* region of the HIV genome, based on previously reported techniques (38, 39). Simultaneous detection of *psi* and *env* was only possible for 19/39 (48.72%) of HIV+ participants. For intact HIV DNA no significant differences were found between participants treated in Fiebig II-III and Fiebig IV-VI (T0: p=0.15, median 10549 and 3594 copies/10^6^ CD4 T cells; UD: p=0.43, median 136 and 288 copies/10^6^ CD4 T cells; UD+1: p=0.11, median 62.60 and 34.40 copies/10^6^ CD4 T cells respectively) (**Figure 2B**). As expected, intact HIV DNA was significantly (p<0.001) lower than total HIV DNA at all timepoints (**Table 2**, **Figure 2C**). After ART initiation, a significant decline in total (p<0.001) and intact (p=0.032) HIV DNA was observed between T0 and UD. During viral suppression a further decline of the HIV reservoir was observed between UD and UD+1 both in total as in intact HIV DNA (p=0.0012 total HIV DNA, p=0.032 intact HIV DNA).

We observed a large interparticipant variation in terms of reservoir composition within our cohort (**Figure 2D**). The ratio of intact to total HIV DNA was significantly higher at UD than at UD+1 (p=0.0081, 76% [IQR 47-81.8] and 42.4% [IQR 29.3-60.9] respectively). It has been established that during the first year after ART initiation, there is a rapid decay of the viral reservoir, after which the decay rate slows down (40). Paired longitudinal analysis of the HIV reservoir in our cohort revealed a more pronounced fold decrease of the intact reservoir versus the total reservoir in the period between UD and UD+1 (p=0.013), which spanned a median of 379 days [IQR 359-462] (**Figures 2E, F**).

### 2.4 Acute HIV-1 infection is characterized by a pleiotropic release of systemic soluble mediators with distinct temporal patterns

**Figure 3:**
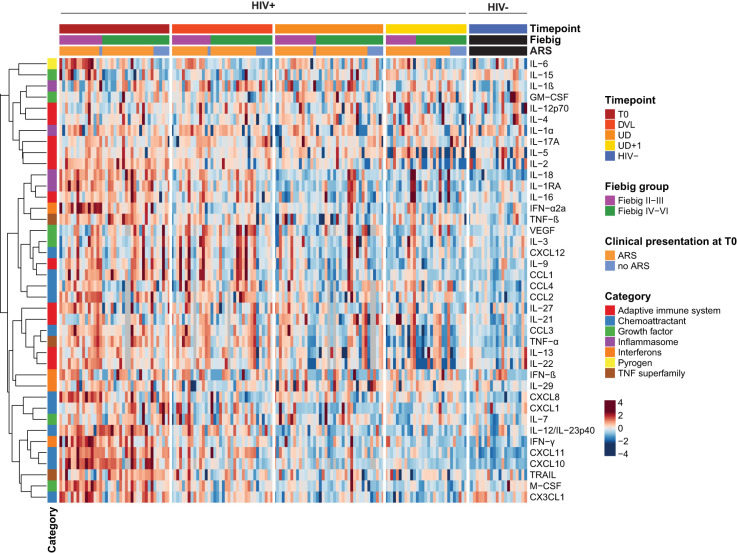
Acute HIV-1 infection is characterized by a pleiotropic release of systemic soluble mediators with distinct temporal patterns. Heatmap displaying individual batch-corrected log2 transformed, row-scaled levels of soluble mediators for early treated HIV-positive (HIV+) and HIV-negative (HIV-) participants per timepoint. Columns represent plasma samples at the different timepoints. Rows represent soluble mediators and are clustered with Spearman's rank correlation coefficient as distance metric.

In order to establish in-depth inflammatory profiles during AHI and longitudinally after introduction of ART, we longitudinally measured plasma concentrations of 40 soluble mediators consisting of adaptive immune system cytokines, chemoattractants, growth factors, inflammasome-related mediators, interferons, pyrogens and TNF superfamily cytokines. Representation of all measured plasma samples in a heatmap illustrates the main trends across the four timepoints, but also highlights the large inter-individual variability (**Figure 3**). During acute HIV infection, a pleiotropic increase of plasma cytokines and chemokines was observed. Compared to the HIV- group, a significantly higher expression of type II interferon (IFN-γ), and type II IFN stimulated chemokines (CXCL10, CXCL11), inflammasome-associated cytokines (IL-18, IL-1RA), type I IFN (IFN-α2a), tumor necrosis factor (TNF) family cytokines (TNF-α, TNF-β, TRAIL), monocyte chemoattractants (CCL1, CCL2, CCL3, CCL4), monocyte secreted chemokines (CXCL8), Th1 polarizing cytokines (IL-12/23p40) and lymphocyte growth factors (IL-2, IL-7) was found on the T0 timepoint (**Figure 3**, **Table 3**). Furthermore, our data confirmed the temporal and transient induction of soluble mediators during AHI as described previously (1, 41), with participants in the earlier phase of AHI (Fiebig stage II-III) showing higher levels of IFN-α2a (p<0.001), IL-15 (p<0.001), IL-6 (p=0.0014), CXCL8 (p=0.0018), IFN-γ (p=0.003), CCL2 (p=0.02), IL-27 (p=0.031)), M-CSF (p=0.045), and CCL4 (p=0.047), as compared to those in a later phase of AHI (Fiebig stage IV-VI). (**Figure 3**, **Supplementary Figure 2A**). Plasma concentrations of IL-22 showed a similar trend, but did not reach significance (p=0.09). Some markers, such as IL-27, IL-6, and IL-22 normalized spontaneously by the later Fiebig stages. Notably, during the later phase of AHI, the concentrations of IL-15 appeared lower compared to the levels observed in the HIV- group, although this trend was not statistically significant (p=0.08).

**Table 3:** Plasma concentrations of soluble mediators.

Soluble mediator	HIV-	HIV+			
		T0	DVL	UD	UD+1
**CXCL10**	134.45 [110.32-195.58]	1301.63 [669.98-1929.78]***	296.39 [187.21-459.01]***	241.07 [179.2-346.27]**	206.29 [180.19-282.69]*
**CXCL11**	13.67 [11.06-16.77]	74.45 [53.9-92.76]***	34.24 [25.49-75.77]***	25.86 [16.73-42.41]***	23.08 [16.83-33.81]**
**IL-18**	342.93 [274.67-373.91]	1083.95 [735.46-1391.15]***	637.38 [477.51-927.1]***	451.34 [391.16-560.33]**	505.26 [385.21-597.69]***
**IFN-γ**	3.23 [2.08-6.35]	15.37 [8.98-53.83]***	5.91 [4.11-11.02]*	5.24 [3.25-8.66]*	4.35 [3.08-7.62]
**CXCL8**	1.43 [1.32-1.75]	3.49 [2.37-4.68]***	1.61 [1.36-2.3]	2.18 [1.59-2.78]*	2.12 [1.46-2.65]*
**TNF-α**	1.19 [0.94-1.76]	3.24 [2.08-4.33]***	2.52 [1.7-4.04]***	1.86 [1.11-3.08]	0.99 [0.74-2.06]
**CCL1**	7.77 [6.66-8.58]	13.46 [10.4-16.3]***	10.38 [7.78-13.49]***	8.58 [6.6-10.67]	8.51 [7.16-10.24]
**IFN-α2a**	0.41 [0.32-0.6]	0.82 [0.57-2.27]***	0.38 [0.33-0.54]	0.44 [0.32-0.52]	0.38 [0.25-0.62]
**IL-12/IL-23p40**	145.63 [80.93-199.62]	281.3 [218.96-348.45]***	192.9 [132.8-272.46]*	111.57 [84.73-164.24]	107.55 [84.69-137.03]
**IL-1RA**	125.62 [75.81-167.15]	282.58 [168.16-436.62]***	141.41 [105.47-248]	95.42 [72.74-144.13]	88.52 [70.22-149.34]
**CCL2**	70.63 [65.12-77.09]	101.57 [87.47-144.46]***	97.21 [73.51-116.77]**	88.15 [68.2-105.46]	80.16 [76.07-107.84]**
**CCL3**	18.13 [13.91-21.93]	34.1 [27.92-45.98]***	28.95 [21.18-48.07]**	24.88 [16.57-36.13]	19.55 [8.69-31.96]
**IL-9**	0.36 [0.28-0.51]	0.71 [0.47-1.12]***	0.55 [0.42-1.07]**	0.38 [0.27-0.58]	0.33 [0.26-0.47]
**TNF-β**	0.36 [0.25-0.4]	0.48 [0.41-0.63]**	0.39 [0.34-0.46]	0.31 [0.21-0.43]	0.38 [0.3-0.51]
**IL-2**	0.18 [0.08-0.7]	0.48 [0.35-0.88]**	0.27 [0.19-0.35]	0.22 [0.1-0.34]	0.1 [0.02-0.41]*
**IL-7**	0.9 [0.7-1.35]	1.57 [1.03-2.14]**	1.07 [0.73-1.87]	1.28 [0.95-1.75]*	1.12 [0.95-1.44]
**TRAIL**	115.4 [98.85-133.3]	152.46 [129.38-169.53]**	118.63 [101.16-141.17]	115.15 [100.63-136.44]	122.84 [93.13-136.98]
**IL-6**	0.49 [0.32-0.75]	0.79 [0.4-1.48]*	0.5 [0.38-0.81]	0.7 [0.38-1.02]	0.49 [0.37-0.96]
**CCL4**	28.83 [24.97-35.65]	39.76 [32.07-50.27]*	43.23 [30.32-55.98]**	37.58 [29.26-48.64]	37.25 [28.25-50.2]*
**CXCL12**	1321.86 [1205.46-1709.26]	1655.64 [1359.1-2290.92]*	1606.43 [1305.72-2101.39]*	1433.36 [1220.2-1740.83]	1321.86 [1124.13-1757.61]
**IL-22**	0.7 [0.33-1.26]	1.24 [0.71-3.55]*	0.83 [0.51-2.13]	0.79 [0.4-1.38]	0.56 [0.02-2.28]
**CX3CL1**	7077.9 [5925.38-9463.61]	9082.28 [7082.37-12162.16]*	7085.72 [5522.03-8553.89]	5996.02 [5000.33-6975.31]**	4990.98 [4302.24-6341.54]***
**IL-27**	115.67 [107.21-171.51]	170.39 [107.39-239.16]*	137.53 [109.58-207.05]	128.87 [94.55-198.01]	117.34 [86.42-166.07]
**IL-16**	181.97 [160.21-200.37]	251.63 [202.48-328.48]*	204.73 [175.63-268.95]	151.48 [110.38-220.3]	145.98 [121.13-212.12]
**M-CSF**	13.35 [10.91-15.81]	19.98 [14.43-27.72]*	14.09 [11.29-18.12]	11.12 [9.35-14.06]	10.42 [8.85-12.72]
**CXCL1**	35.08 [26.38-53.04]	50.09 [34-92.36]	32.73 [22.82-70.59]	23.9 [21.06-35.4]	28.43 [20.21-43.54]
**IL-1β**	0.01 [0-0.03]	0.03 [0.01-0.08]	0.05 [0.02-0.16]**	0.02 [0-0.14]*	0.02 [0.02-0.05]*
**VEGF**	21.28 [18.17-27.08]	25.5 [18.5-43.11]	37.31 [21.66-75.25]**	21.29 [15.61-39.17]	18.62 [13.95-26.19]
**IFN-β**	0.18 [0.09-2.59]	1.43 [0.11-5.67]	0.58 [0.04-4.57]	0.21 [0.04-4.56]	0.91 [0.09-2.66]
**IL-13**	2.39 [1.43-4.14]	3.8 [2.5-6.81]	3.51 [2.13-6.41]	3.31 [1.25-5.13]	2.64 [0.66-4.26]
**IL-5**	0.41 [0.07-0.88]	0.32 [0.23-0.73]	0.32 [0.19-0.55]	0.49 [0.18-0.77]	0.44 [0-1.57]
**IL-3**	10.67 [7.86-14.64]	13.88 [8.43-24.75]	13.2 [9.58-29.06]	11.49 [6.65-18.31]	9.3 [6.41-15.81]
**IL-21**	10.33 [7.46-13.27]	12.6 [9.27-19.18]	13.29 [9.54-21.12]	15.39 [9.07-21.12]	13.01 [9.57-18.73]*
**IL-29**	8.62 [7.93-10.65]	11.06 [7.37-15.02]	8.35 [6.59-12.15]	11.43 [7.94-16.49]	10.9 [8.79-12]
**IL-15**	2.54 [2.17-2.73]	2.09 [1.58-3.01]	1.97 [1.67-2.48]	2.28 [1.94-2.71]	2.29 [2.02-2.84]
**GM-CSF**	0.07 [0.04-0.12]	0.06 [0.05-0.1]	0.06 [0.04-0.09]*	0.09 [0.05-0.11]	0.09 [0.05-0.13]
**IL-17A**	1.37 [0.91-2.06]	2.01 [1.21-3.36]	1.59 [1.13-3.69]	1.61 [1.2-2.85]	1.56 [0.78-3.25]
**IL-1α**	0.33 [0.09-0.99]	0.4 [0.13-0.91]	0.36 [0.11-0.83]	0.14 [0.05-0.5]	0.19 [0.06-0.49]
**IL-4**	0.05 [0.03-0.11]	0.06 [0.05-0.07]	0.05 [0.03-0.07]	0.07 [0.04-0.1]	0.05 [0.03-0.08]
**IL-12p70**	0.22 [0.15-0.66]	0.24 [0.19-0.37]	0.29 [0.2-0.42]	0.22 [0.16-0.36]	0.21 [0.13-0.29]

Data are represented as median [IQR] values of batch-corrected plasma concentrations, expressed in pg/mL, in the HIV- control group and HIV+ group at timepoint T0, DVL, UD, and UD+1. Values which differed significantly from the HIV- group are indicated with *, p<0.05; **, p<0.01; ***, p<0.001 and marked in bold. P-values were calculated using a linear mixed regression model with timepoint and batch as fixed predictors.

By comparing the inflammatory profile of HIV+ participants with and without ARS, we identified the soluble mediators contributing most to systemic clinical manifestation. ARS was associated with higher levels of the endogenous pyrogen IL-6 (p=0.021), the inflammasome regulator IL-1RA (p=0.038), and the IFN-γ induced chemokine CXCL10 (p=0.032), compared to participants without ARS. IFN-γ was higher in participants with ARS, compared to the HIV- group (p<0.001), while no statistically significant difference was observed between the control group and the participants without ARS (**Figure 3**, **Supplementary Figure 2B**). In contrast, participants without ARS had higher plasma levels of the neutrophil chemoattractant CXCL1, compared to those with ARS (p=0.033), as well as compared to the HIV- group (p=0.0054). The hematopoietic growth factor IL-7 showed a similar trend.

### 2.5 Early ART initiation cannot prevent the persistence of low-grade systemic inflammation in PLWH

We then assessed the longitudinal course of the inflammatory profile after ART initiation (**Figure 3**, **Table 3**). One month after ART initiation (DVL), most soluble mediators showed a decreasing trend. Despite the detectable plasma viral load, some cytokines and chemokines had completely normalized by this timepoint. Examples are the rapidly induced IL-27, IL-6, IL-22, M-CSF, and IFN-α2a which already showed a normalization or declining pattern between early and late Fiebig stages. In contrast, VEGF showed a delayed peak, with increasing levels one month after AHI diagnosis, and a spontaneous normalization afterwards (**Supplementary Figure 3A**). Interestingly, some soluble mediators did not normalize with suppressive ART, even at the UD+1 timepoint, on average 2 years after treatment initiation: type II IFN induced chemokines CXCL11 (p=0.002) and CXCL10 (p=0.028); inflammasome-related cytokines IL-18 (p<0.001) and IL-1β (p=0.015); chemoattractants CCL2 (p=0.0081), CCL4 (p=0.015), CXCL8 (p=0.035); and IL-21 (p=0.028) (**Supplementary Figure 3B**). On the other hand, CX3CL1, a key regulator of cytotoxic T-cells, was significantly lower at the UD+1 timepoint, compared to the HIV- group (p<0.001), despite an initial upregulation during acute HIV infection. Plasma levels of IFN-γ, the inducer of CXCL11 and CXCL10 showed a gradual decline over time and were still significantly higher than the HIV- group at the UD timepoint (p=0.044), but no longer at the UD+1 timepoint (p=0.13) (**Supplementary Figure 3C**).

### 2.6 The persistent pro-inflammatory signature after early ART correlates to pretreatment inflammation, which is likely driven by viremia

**Figure 4:**
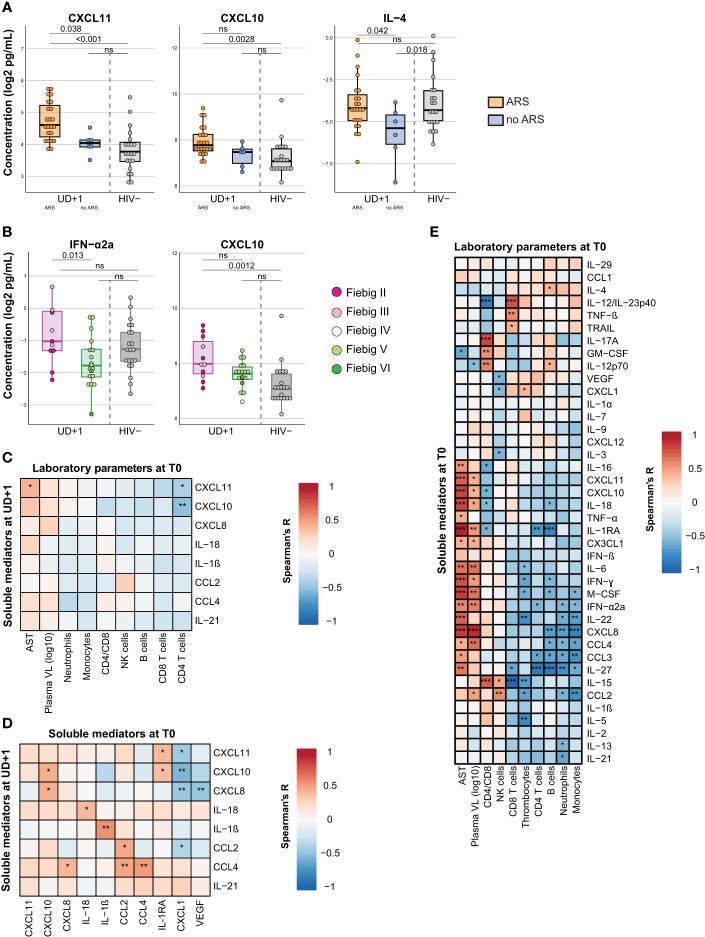
The persistent pro-inflammatory signature after early ART correlates to pretreatment inflammation, which is likely driven by viremia. **(A)** Box plots displaying plasma concentrations of CXCL11, CXCL10, and IL-4 at the UD+1 timepoint in HIV+ participants presenting with (orange) and without (blue) ARS at diagnosis and HIV- participants (grey). P-values were calculated using a linear model with batch as a fixed factor. **(B)** Box plots displaying plasma concentrations of IFN-a2a and CXCL10 at the UD+1 timepoint in HIV+ participants diagnosed and treated in Fiebig stages II-III (purple) and Fiebig stages IV-VI (green) and HIV- participants (grey). Dots are colored according to Fiebig stages. P-values were calculated using a linear model with batch as a fixed factor. **(C, D)** Correlation matrixes between plasma concentrations of soluble mediators during suppressed viremia (UD+1) and laboratory parameters at baseline (T0) **(C)** and plasma concentrations of soluble mediators at baseline (T0) **(D)**. Correlation coefficients and p-values were calculated with Spearman's rank test for pairwise complete observations. Positive and negative correlations are depicted in red and blue, respectively. Significant correlations are indicated by *, p<0.05; **, p<0·01; ***, p<0·001. **(E)** Correlation matrix between plasma concentrations of soluble mediators and laboratory parameters of HIV+ participants during acute HIV infection (T0). Correlation coefficients and p-values were calculated with Spearman's rank test for pairwise complete observations. Positive and negative correlations are depicted in red and blue, respectively. Significant correlations are indicated by *, p<0·05; **, p<0.01; ***, p<0·001. All soluble mediator concentrations were batch-corrected and log2 transformed. ARS, acute retroviral syndrome; AST, aspartate aminotransferase; VL, viral load; ns, not significant.

To understand the contributing factors to the persistent inflammatory profile during viral suppression, we examined differences in pretreatment characteristics. Interestingly, clinical presentation with ARS during acute HIV infection, was associated with higher plasma concentrations of CXCL11 (p=0.038) at the UD+1 timepoint. CXCL10, another type II IFN induced chemokine, showed a similar trend, but did not reach statistical significance (p=0.14) (**Figure 4A**). The comparison to the HIV- group suggests that the persistently elevated levels of CXCL11 and CXCL10 at UD+1 were mainly driven by the participants presenting with ARS during AHI. Plasma concentrations of IL-4 at UD+1 were marginally lower in participants without ARS during AHI, compared to those with ARS (p=0.042) and the HIV- group (p=0.018), but were generally very low. Furthermore, we looked whether earlier ART initiation, in Fiebig stages II-III versus Fiebig stages IV-VI had an impact on the levels of soluble mediators under stable, suppressive ART. ART initiation during the earlier Fiebig stages was associated with higher levels of IFN-α2a (p=0.013) at UD+1, compared to ART initiation in later Fiebig stages (**Figure 4B**). Levels of CXCL10 at UD+1 showed a similar, but non-significant trend (p=0.088). Only the participants diagnosed in Fiebig stages II-III showed a significant difference in plasma CXCL10 with the HIV- group (p=0.0012), not the participants diagnosed in the later Fiebig stages (p=0.19). Collectively, these findings suggest that events early in the course of HIV infection have a lasting impact on low-grade inflammation.

When specifically examining the correlations between the identified group of persistently elevated cytokines and chemokines at UD+1 and the pretreatment immunological parameters at T0, we found that the CD4 nadir during acute HIV infection correlated inversely with plasma concentrations of CXCL11 and CXCL10 at UD+1 (**Figure 4C**). Generally, however, the most significant positive correlations between the persistently elevated levels of CXCL10, IL-18, IL-1β, CCL2 and CCL4 (UD+1) were found with their pretreatment levels during AHI (T0) (**Figure 4D**). During acute HIV-infection, these mediators clustered together and showed a positive correlation to plasma viral load and levels of aspartate aminotransferase, suggesting that they might be driven by viral replication. (**Figure 4E**).

In contrast, CXCL1 at T0 showed an inverse correlation with the pro-inflammatory signature at UD+1, with negative correlations with CXCL11, CXCL10, CXCL8 and CCL2 (**Figure 4D**). Remarkably, among the cellular laboratory parameters, no clear immunological signature during viral suppression was found to correlate with the identified group of persistently elevated cytokines. Only absolute NK cell counts showed a positive correlation with CCL2 and CD4/CD8 ratio correlated with CXCL10 (**Supplementary Figure 4**).

### 2.7 The CD4/CD8 ratio and time to viral suppression are correlated with distinct soluble mediators following acute HIV infection

**Figure 5:**
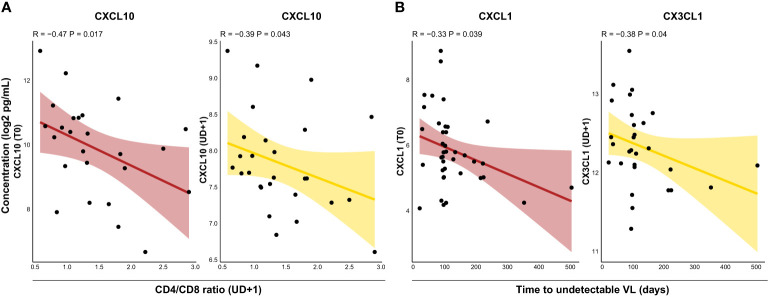
The CD4/CD8 ratio and time to viral suppression are correlated with distinct soluble mediators following acute HIV infection. **(A)**, Correlations between CD4/CD8 ratio during suppressed viremia (UD+1) and plasma concentrations of CXCL10 at T0 (burgundy) and UD+1 (yellow). **(B)** Correlations between time to undetectable HIV viral load and plasma concentrations of CXCL1 at T0 (burgundy) and CX3CL1 at UD+1 (yellow). All soluble mediator concentrations were batch-corrected and log2 transformed. Correlation coefficients and p-values were calculated with Spearman's rank test. VL, viral load.

CD4/CD8 ratio is often used as a proxy for immune activation and residual inflammation in PLWH and correlates with clinical outcomes (42, 43). Indeed, we found inverse correlations between the CD4/CD8 ratio during viral suppression and plasma levels of CXCL10 both at T0 (R=-0.47, p=0.017) and UD+1 (R=-0.39, p=0.043) (**Figure 5A**). Time to viral suppression is another parameter which is often taken into account in assessing immunovirological outcomes. Because of the potent integrase strand transfer inhibitor (INSTI) regimens used in our cohort, rapid viral suppression was obtained in most HIV+ participants after approximately 100 days. However, in some participants time to viral suppression was notably longer, raising the question whether the inflammatory profile during AHI or during viral suppression could be associated with this phenomenon. Interesting, albeit modest correlations were found between time to viral suppression and T0 plasma levels of CXCL1 (R=-0.33, p= 0.039) and UD+1 levels of CX3CL1 (R=-0.38, P=0.04) (**Figure 5B**).

### 2.8 Inflammatory profiles in the course of acute HIV infection show bidirectional correlations to the viral reservoir

**Figure 6:**
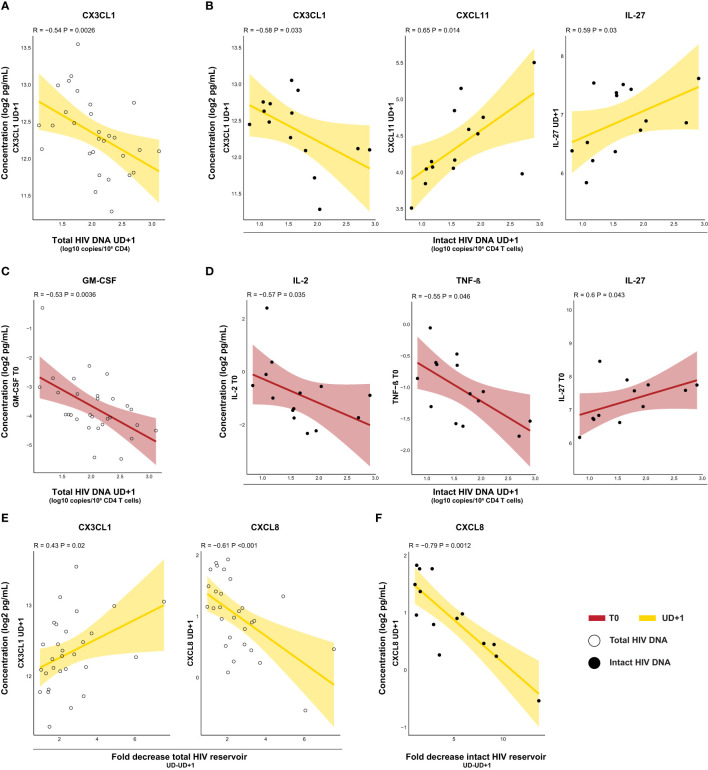
Inflammatory profiles in the course of acute HIV infection show bidirectional correlations to the viral reservoir. **(A, B)** Correlations of HIV reservoir at UD+1 in relation with soluble mediators at UD+1. Inverse correlation of total HIV DNA with plasma concentrations of CX3CL1 **(A)** and correlation of intact HIV DNA **(B)** with plasma concentrations of CX3CL1, CXCL11, and IL-27. **(C, D)** Correlations of HIV reservoir at UD+1 with soluble mediators at T0. Inverse correlation of total HIV DNA in relation with plasma concentrations of GM-CSF **(C)** and correlation of intact HIV DNA **(D)** in relation with plasma concentrations of IL-2 and TNF-β (inverse) and IL-27. **(E)** Correlations of fold decrease in total HIV DNA between UD and UD+1 in relation with plasma concentrations of CX3CL1 and CXCL8 (inverse) at UD+1. **(F)** Correlations of fold decrease in intact HIV DNA between UD and UD+1 in relation with plasma concentrations of CXCL8 (inverse) at UD+1. All concentrations were batch-corrected and log2 transformed. Correlation coefficients and p-values were calculated with Spearman's rank test.

Next, we assessed the relationship between the plasma inflammatory profile and HIV reservoir. Immunological signaling molecules might not only be produced in response to cells harboring HIV, their presence might also impact homeostatic maintenance or clearance of the viral reservoir. We first examined correlations between the HIV reservoir and contemporaneous soluble mediator levels at UD+1. In general, more correlations were found with the intact reservoir, than the defective or total reservoir (**Supplementary Figure 5**). CX3CL1 showed an inverse correlation with total HIV DNA (R=-0.54, p-p=0.0026) and intact HIV DNA (R=-0.58, p=0.033) (**Figures 6A, B**). Interestingly, intact HIV DNA exhibited a positive correlation with levels of CXCL11 (R=0.65, p=0.014), one of the chemokines which remained persistently elevated at UD+1 (**Figure 6B**). In addition, levels of IL-27, a pleiotropic cytokine with both pro-inflammatory and immunosuppressive capabilities, were also correlated with higher intact HIV DNA (R=0.59, p=0.03) (**Figure 6B**). To assess whether the inflammatory profile during acute HIV infection had a lasting impact on the viral reservoir during viral suppression, we correlated both total and intact HIV DNA with pretreatment levels of soluble mediators. Total HIV DNA was inversely correlated with levels of GM-CSF at T0 (R=-0.53, p=0.0036) (**Figure 6C**). Intact HIV DNA was inversely correlated with IL-2 (R=-0.57, p=0.035) and TNF-β (R=-0.53, p=0.0036) and positively associated with IL-27 (R=0.6, p=0.043) (**Figure 6D**). The fold decrease in total HIV DNA was positively associated with levels of CX3CL1 (R=0.43, p=0.02) during viral suppression (UD+1) (**Figure 6E**). Plasma levels of the pro-inflammatory chemokine CXCL8 at UD+1 showed a strong inverse correlation with both the fold decrease in total (R=-0.61, p<0.001) and intact reservoir (R=-0.79, p=0.0012) (**Figures 6E, F**).

## 3 Discussion

To evaluate the impact of early ART on systemic inflammation and the interplay with the HIV reservoir, we assessed the plasma inflammatory profile longitudinally during and after AHI and integrated this with clinical presentation during AHI, immunological parameters and in-depth characterization of the HIV reservoir.

Our results confirm previous observations that ART initiated during AHI is able to rapidly abrogate the characteristic cytokine storm, but fails to normalize some of the plasma inflammatory markers which are induced during the acute phase of infection, even after long-term viral suppression (24–28). Importantly, we identified eight soluble mediators which remained elevated in plasma of very early treated HIV+ individuals compared to HIV-negative controls. Some of these soluble mediators have been described in one or more other AHI cohorts, such as IFN-γ-induced CXCL10 (24, 26, 27) and CXCL11 (24), and CXCL8 (44), CCL2 (24, 26, 27, 44) and CCL4 (44) chemokines. The main cellular source of these chemokines are activated monocytes and macrophages. Moreover, CXCL10, CXCL11, CCL2, and CCL4 also function as monocyte chemoattractants and CXCL8 is a neutrophil chemoattractant. The validation of these findings in our cohort underscores a shared pattern of myeloid compartment activation, consistently observed in early-treated PLWH across diverse geographic and demographic contexts. In contrast, our study did not observe elevations in some previously identified mediators, such as TNF-α and IL-6 (26). Furthermore, we observed consistently elevated levels of IL-18 and IL-1β, two cytokines not previously identified in AHI cohorts. It is noteworthy that these two cytokines have been underrepresented in the soluble mediator panels used in other studies. Only Bordoni et al. included these markers and they did not report a significant difference between PLWH treated during AHI and their relatively small HIV- control group (n=7) (44). Evidence from chronic HIV infection, however, suggests that plasma IL-18 remains elevated, despite ART (45–48). Circulating levels of IL-1β in plasma are generally low and are difficult to measure, requiring high-sensitivity immunoassays, but upregulation of the IL-1β pathway in monocytes has recently been illustrated in a large cohort of PLWH, with evidence for increased innate cytokine responses as a result of trained immunity (48). Our data suggest that even when ART is initiated during the earliest stages of infection, there is persistent inflammasome activation. This implies that the proposed transcriptional and functional reprogramming of monocytes either occurs very early during AHI or persists as an ongoing process during suppressive ART. Both IL-1 β and IL-18 can induce downstream activation of a wide array of innate and adaptive immune cells, triggering the release of other pro-inflammatory mediators such as CCL3, CCL4, and IFN-ɣ. The latter, in turn, can induce the production of CXCL10 and CXCL11. Consequently, inflammasome activation might play a pivotal role in sustaining low-grade inflammation.

Pretreatment levels of nearly all persistently upregulated inflammatory markers correlated with plasma viral load during AHI, reinforcing similar findings by Teigler et al. (24) We also found a high level of correlation between these soluble mediators and their pretreatment levels, consistent with the findings of Gandhi et al. in a chronic HIV cohort, which demonstrated that high levels of inflammation before ART initiation correlate with high levels of inflammation during suppressive ART (49). Likewise, plasma concentrations of CXCL10 and CXCL11 during suppressive ART were higher in individuals who presented with clinical signs and symptoms of ARS during AHI. This suggests that the degree of immune activation during AHI, potentially driven by the virus, not only impacts viral setpoint, as previously demonstrated (10), but might also exert a lasting impact on systemic inflammation. Intriguingly, plasma concentrations of the neutrophil chemoattractant CXCL1 during AHI were higher in participants without ARS presentation and showed a strong negative correlation with the persistent pro-inflammatory signature during viral suppression. Furthermore, higher pretreatment levels of CXCL1 were associated with a faster time to viral suppression. These remarkable findings stand in contrast to studies which identify CXCL1 as a driver for HIV replication (50) and suggest a context-dependent function for CXCL1. The precise role of neutrophils during acute HIV infection and its long-term impact on systemic inflammation require further elucidation.

To investigate the crosstalk between viral factors and inflammation more in-depth, we measured both total, intact and defective HIV reservoir longitudinally during viral suppression over a period of 1 year. While defective proviruses may lack replication competence, they can still exhibit transcriptional or even translational activity, thereby potentially contributing to immunological processes (16, 17, 51). Therefore, we examined to what extent the intact and defective proviral reservoir relate to inflammation. We found that the inflammatory profile during viral suppression was more associated to the intact than the defective reservoir, which could explain the limited number of associations between reservoir and plasma inflammatory markers documented in previous AHI studies, which did not assess intactness of the reservoir (24, 25). This emphasizes the value of intactness measurements in future research focused on inflammation and the HIV reservoir. Two pro-inflammatory chemokines, which were persistently elevated, CXCL11 and CXCL8, were associated with a larger intact reservoir size and slower decay of the reservoir, respectively. This highlights that there is crosstalk between low-grade inflammation and reservoir dynamics. Notably, CXCL11 and other IFN-ɣ induced chemokines have been linked to T-cell dysregulation (52). However, it is important to note that our results do not allow us to establish causality within this relationship. Several hypotheses are plausible: the reservoir could be a driver of low-grade inflammation, inflammation-related immunopathology could reduce viral reservoir clearance or both observations might be independently influenced by another common underlying factor, possibly impacted by variations in viral and host factors. Interestingly, we also found soluble mediators which exhibited a favorable association with the reservoir. Higher levels of the chemokine CX3CL1 during viral suppression were associated with faster decay of the intact reservoir and a shorter time to viral suppression. CX3CL1, also known as fractalkine, is mainly produced by endothelial cells and exists in a secreted and membrane-bound form. It is a mediator of cellular adhesion and acts as a key regulator of cytotoxic T cells. We observed a remarkable longitudinal pattern of CX3CL1, with lower plasma levels in HIV+ versus HIV- individuals during viral suppression, despite an initial upregulation during acute HIV infection. A similar declining trend has been shown previously in early and late chronic ART-naïve HIV+ individuals (24). Recent *in-vitro* data on astrocytes suggest that HIV limits the capacity of astrocytes to express CX3CL1 by blocking the interaction between NF-κB and the CX3CL1 promotor (53). This prompts the question whether CX3CL1 contributes to the clearance of the viral reservoir or whether production of CX3CL1 itself is downregulated by active components of the reservoir. Evidence for the first hypothesis can be drawn from the cancer field, in which anti-cancer properties have been attributed to CX3CL1. Soluble CX3CL1 has been associated with better prognosis in colorectal cancer (54), has demonstrated antitumor effects in murine lung (55) and hepatocellular cancer models (56) and was identified as a predictor for response to immune checkpoint blockade in patients with non-small-cell-lung cancer (57). Mechanistically, these activities may be mediated through chemotaxis of T cells and NK cells, as well as the maintenance of a potent effector memory cytotoxic T cell population (58). Furthermore, soluble CX3CL1 has been shown to increase myeloid phenotypic diversity, leading to an increase in the monocytic lineage and a decrease in granulocytes (57).

The HIV reservoir was not only associated with contemporaneous soluble mediators, but also with the inflammatory profile during the acute phase of infection. During AHI, IL-27 was the only soluble mediator displaying a positive association with long-term viral reservoir. Elevated levels of this cytokine have previously been associated with a larger HIV reservoir and a reduction in magnitude and breadth of HIV-specific cytotoxic T-cell activity (59). Conversely, higher levels of GM-CSF and IL-2 during AHI were linked to a smaller reservoir size. Historically, systemic administration of both growth factors have been studied as add-on therapy for ART during chronic HIV infection in an attempt to boost cell-mediated immunity (60–63). GM-CSF stimulates hematopoiesis in both myeloid and lymphoid lineages, enhances antigen presentation by macrophages and may reduce the infectability of macrophages by downregulation of CCR5 expression (64). While GM-CSF has demonstrated positive effects on CD4 T-cell counts and plasma viral loads, its high cost, side-effects, and rapid improvements in the field of antiretrovirals have decreased the scientific interest in this field (61). IL-2 promotes lymphocyte proliferation and differentiation, and its secretion during AHI has been associated with better CD4 T-cell maintenance (65). Existing evidence suggests that IL-2 in combination with ART, increases CD4 T-cells counts in PLWH; however, systemic administration has also been associated with a considerable increase in adverse events (63). The effect of these two growth factors on the HIV reservoir has not been extensively studied in past research. Our novel finding that higher levels of GM-CSF and IL-2 during AHI have favorable effects on the reservoir size, raise the question whether carefully-timed co-administration of these growth factors with ART during acute HIV-infection or during therapeutic vaccination might yield a positive effect on the viral reservoir.

Our study presents several limitations. Firstly, there is a lack of epidemiological diversity within the HIV+ study population. The predominance of white MSM in the ACS-cohort is a reflection of the demographics of the population diagnosed with AHI in Belgium. However, this may limit the generalizability of our findings to other settings. Variations in inflammatory profiles in PLWH have been reported based on geographical location, HIV subtype, and gender (66, 67). The underrepresentation of women in our study may contribute to the observed differences with other AHI cohorts, as research has illustrated that women with primary HIV infection tend to have lower peak viral loads, but higher levels of subsequent immune activation, and worse clinical outcomes (68, 69). Another limitation relates to the fact that our HIV- control group could not be matched for risk-behavior. Research by Novelli et al. has shown that non-HIV-related lifestyle factors may significantly influence the inflammatory profile observed in early treated PLWH (25). A third limitation of our study pertains to the measurement of the intact reservoir, which was performed with a PCR-based multiplex assay, based on the classical intact proviral DNA assay (IPDA) (39). A measurement for intactness could only be obtained in 48% (19/39) of the HIV+ participants because of assay failure due to subtype diversity and genetic polymorphism, which is a known limitation of this technique (70). This reduced number of measurements might have limited our statistical power to explore associations between the intact reservoir and the inflammatory profile. However, it is worth noting that more correlations were found with intact than total reservoir, reaffirming the robustness of these associations. Additionally, IPDA is known to overestimate the size of the intact reservoir since it does not account for deletions present between the *env* and *psi* region. This might have contributed to the large intact reservoir measurement obtained in some study participants (39). Lastly, our study solely focused on inflammatory mediators in plasma and the viral reservoir in blood. It is well known that the majority of the HIV reservoir is located in lymphoid organs, such as gut and lymph nodes and that the tissues are a major site for HIV-related immunopathology (71). Furthermore, the quintessence of cytokines, chemokines, and growth factors is their function as messenger molecules which can steer or inhibit immune processes throughout the entire body. Understanding how systemic inflammation is related to viral reservoir dynamics in lymphoid tissues and the extent to which this is a reflection of the local inflammatory milieu within tissues remains a crucial area for further investigation. The use of high-plex *in-situ* profiling of the inflammatory milieu within anatomical reservoir sites could provide valuable insights to this field (72).

In conclusion, this study demonstrates that suppressive ART, initiated during AHI is unable to prevent low-grade inflammation characterized by activation of the myeloid compartment. Our research identifies key molecules in this pro-inflammatory signature and uncovers the involvement of inflammasome-related pathways. Furthermore, we identify individual soluble mediators produced during acute HIV infection which show long-term associations to the HIV reservoir at viral suppression. This underscores the importance of inflammatory milieus during the earliest stages of HIV infection. Our research demonstrates that CXCL11 and CXCL8, two persistently elevated pro-inflammatory chemokines, are associated to larger a reservoir size and reduced decay, implying a potential association between low-grade inflammation and unfavorable virological outcomes In addition, we show that higher levels of the chemokine CX3CL1 during viral suppression were related to a smaller reservoir size, faster decay of the total reservoir and a shorter time to viral suppression. Taken together, in this study we identify novel inflammatory markers of reservoir size and composition after acute HIV infection, which provide interesting hypotheses for follow-up studies investigating mechanistic pathways or novel therapeutic targets.

## 4 Materials and methods

### 4.1 Study participants

All HIV-positive samples and data were obtained from the ongoing ACS-study, “Accurate staging of immuno-virological dynamics during acute HIV-1 infection”, a multicentric prospective AHI cohort in Belgium coordinated at the HIV Cure Research Center at Ghent University (ClinicalTrials.gov identification NCT03449706). Inclusion criteria were adult age (18-65 years) and documented acute HIV infection defined as a new diagnosis of HIV with clinical symptoms of acute seroconversion, a negative screening test within the past 6 months or the presence of a risk contact within the last 3 months, and negative or incomplete line immunoassay. Exclusion criteria were recent use of immunomodulatory drugs and active hepatitis C infection. Participants were included at Ghent University Hospital, AZ Sint-Jan Bruges, CHU Saint-Pierre, and UZ Brussels. Participants were included during AHI and started on ART within one week. Blood draws were performed before ART initiation (T0) and longitudinally during follow-up. Leukapheresis was performed after suppression of plasma viral load (UD) and one year later (UD+1).

HIV-negative control participants were included at Ghent University Hospital in the VIM-study, “Virological, immunological and microbiome monitoring platform in HIV-infected populations”. HIV-testing was negative in all participants.

### 4.2 Clinical and laboratory parameters

Clinical and laboratory parameters were assessed at every study visit. Plasma viral loads were measured using the Cobas® HIV-1 test (Roche Diagnostics, Basel, Switzerland). Classification of AHI was performed as described by Fiebig et al (73), using results of plasma HIV RNA determination, fourth-generation immunoassay (VIDAS DUO), and INNO-LIA HIV I/II Score (Fujirebio).

Acute retroviral syndrome was defined based on the presence of fever or ≥2 of the following signs and symptoms around the time of the positive HIV diagnostic test: adenopathy, night sweating headache, fatigue, myalgia, arthralgia, pharyngitis, oral ulcer, genital ulcer, weight loss, anorexia, nausea/vomiting, diarrhea, odynophagia, skin rash, oral candidiasis, vaginal candidiasis, or other neurologic symptoms.

Study data were collected and managed using REDCap electronic data capture tools hosted at Gent University Hospital (74, 75).

### 4.3 Sample collection

Whole blood was collected from study participants by blood draw or by leukapheresis. Plasma was collected after peripheral blood mononuclear cell (PBMC) separation, with Lymphoprep™ (# 07851, Stemcell technologies) in Leucosep™ tubes (# 227290, Greiner Bio-One), aliquoted and stored at -80°C. PBMCs were collected and cryopreserved in FCS/DMSO 10% in liquid nitrogen.

### 4.4 Soluble mediator measurements

Plasma samples were thawed on ice and quantified using four Mesoscale Discovery U-Plex electrochemiluminescent immunoassay 10-plex panels (#K15067L-1, MSD) according to the manufacturer’s instructions in two batches. The following 40 analytes were included: CX3CL1, GM-CSF, CXCL1, CCL1, IFN-α2a, IFN-β, IFN-γ, IL-12p40/IL-23p40, IL-12p70, IL-13, IL-15, IL-16, IL-17A, IL-18, IL-1RA, IL-1α, IL-1β, IL-2, IL-21, IL-22, IL-27, IL-29, IL-3, IL-4, IL-5, IL-6, IL-7, CXCL8, IL-9, CXCL10, CXCL11, CCL-2, M-CSF, CCL3, CCL4, CXCL12, TNF-α, TNF-β, TRAIL, VEGF. Analyte concentrations were inferred from standard curves. Values below the fit curve were replaced by the square root of the minimum value per analyte per run. A linear mixed model with participant ID as random factor and batch and timepoint as fixed factors was used to correct for batch effects between the two batches.

### 4.5 Total, intact and defective HIV DNA measurements

CD4+ T cells were isolated from fresh or cryopreserved PBMCs using EasySep™ Human CD4+ T Cell Isolation Kit (#17952, StemCell technologies), after which genomic DNA was extracted using QIAamp DNA Mini Kits (#51304, Qiagen). Total and intact HIV DNA were measured simultaneously with the Rainbow proviral DNA digital PCR assay (38), using the QIAcuity digital PCR platform. This multiplex assay includes RU5 primers and probes for total HIV DNA detection (76) and the *psi* and *env* primers and probes of the IPDA assay (39). *RPP30* was used as a reference gene (38). Intactness was quantified based on the detection of *env* and *psi* and corrected for shearing using a DNA Shearing Index (DSI) correction, as previously described (39). Defective HIV DNA was calculated by subtracting intact from total HIV DNA.

### 4.6 Statistics

Categorical variables are presented as frequency counts and percentages, continuous variables are presented as medians and interquartile ranges. Categorical data between groups were compared using Chi square tests. Unpaired continuous data between groups were compared using Mann Whitney U tests (2 groups). Paired continuous data were compared using Wilcoxon signed-rank tests (2 groups) or Friedman tests with *post-hoc* Dunn tests (>2 groups). Correlations were calculated by two-sided Spearman’s rank tests for pairwise complete observations. P values <0.05 were considered statistically significant. Due to sample size limitations and the exploratory nature of this study, no correction for multiple testing was applied. All statistics were performed using R-4.2.1 software.

### 4.7 Study approval

The ACS-study (early treated HIV-positive participants) and VIM-study (HIV-negative participants) were approved by the Ethics Committee of Ghent University Hospital, Belgium (BC-00812 and BC-01373, respectively). All participants provided written informed consent.

## Data availability statement

The original contributions presented in the study are included in the article/**Supplementary Material**, further inquiries can be directed to the corresponding author.

## Ethics statement

The studies involving humans were approved by ethics committee of Ghent University Hospital, Belgium. The studies were conducted in accordance with the local legislation and institutional requirements. The participants provided their written informed consent to participate in this study.

## Author contributions

JDC: Conceptualization, Formal analysis, Investigation, Resources, Visualization, Writing – original draft, Writing – review & editing. M-ADS: Conceptualization, Resources, Writing – review & editing. VM: Investigation, Writing – review & editing. CV: Investigation, Writing – review & editing. SV: Resources, Writing – review & editing. SA: Resources, Writing – review & editing. CN: Resources, Writing – review & editing. SDW: Funding acquisition, Resources, Writing – review & editing. SG: Supervision, Writing – review & editing. LV: Conceptualization, Funding acquisition, Resources, Supervision, Writing – review & editing.
