# Supplementary material for: Longitudinal patterns of inflammatory mediators after acute HIV infection correlate to intact and total reservoir
Source: Front Immunol. 2024 Jan 5;14:1337316. doi: 10.3389/fimmu.2023.1337316 (PMC10796502; doi:10.3389/fimmu.2023.1337316)
Supplement: Supplementary file 1 [file DataSheet_1.pdf]

Supplementary materials

Supplementary table

Supplementary table 1: abbreviation list of soluble mediators

**Soluble mediator**		**Alias**	
**CX3CL1**	C-X3-C motif chemokine ligand 1	Fractalkine	
**GM-CSF**	granulocyte-macrophage colony-stimulating factor		
**CXCL1**	C-X-C motif chemokine ligand 1	GROa	growth-regulated alpha protein
**CCL1**	C-C motif chemokine ligand 1	I-309	
**IFN-α2a**	interferon alpha 2a		
**IFN-β**	interferon beta		
**IFN-γ**	interferon gamma		
**IL-12/IL-23p40**	interleukin 12/interleukin 23 p40		
**IL-12p70**	interleukin 12p70		
**IL-13**	interleukin 13		
**IL-15**	interleukin 15		
**IL-16**	interleukin 16		
**IL-17A**	interleukin 17 A		
**IL-18**	interleukin 18		
**IL-1RA**	interleukin 1 receptor antagonist		

**IL-1α**	interleukin 1 alpha		
**IL-1β**	interleukin 1 beta		
**IL-2**	interleukin 2		
**IL-21**	interleukin 21		
**IL-22**	interleukin 22		
**IL-27**	interleukin 27		
**IL-29**	interleukin 29	IFN-λ	interferon lambda
**IL-3**	interleukin 3		
**IL-4**	interleukin 4		
**IL-5**	interleukin 5		
**IL-6**	interleukin 6		
**IL-7**	interleukin 7		
**CXCL8**	C-X-C motif chemokine ligand 8	IL-8	interleukin 8
**IL-9**	interleukin 9		
**CXCL10**	C-X-C motif chemokine ligand 10	IP-10	interferon gamma-induced protein 10
**CXCL11**	C-X-C motif chemokine ligand 11	ITAC	interferon-inducible T cell alpha chemoattractant
**CCL2**	C-C motif chemokine ligand 2	MCP-1	monocyte chemoattractant protein-1
**M-CSF**	macrophage-colony stimulating factor		
**CCL3**	C-C motif chemokine ligand 3	MIP-1α	macrophage inflammatory protein-1 alpha

**CCL4**	C-C motif chemokine ligand 4	MIP-1β	macrophage inflammatory protein-1 beta
**CXCL12**	C-X-C motif chemokine ligand 12	SDF-1	stromal cell-derived factor 1
**TNF-α**	tumor necrosis factor alpha		
**TNF-β**	tumor necrosis factor beta		
**TRAIL**	TNF-related apoptosis-inducing ligand		
**VEGF**	vascular endothelial growth factor		

List of abbreviations of soluble mediators used in this manuscript and their most commonly used alias.

Supplementary Figures

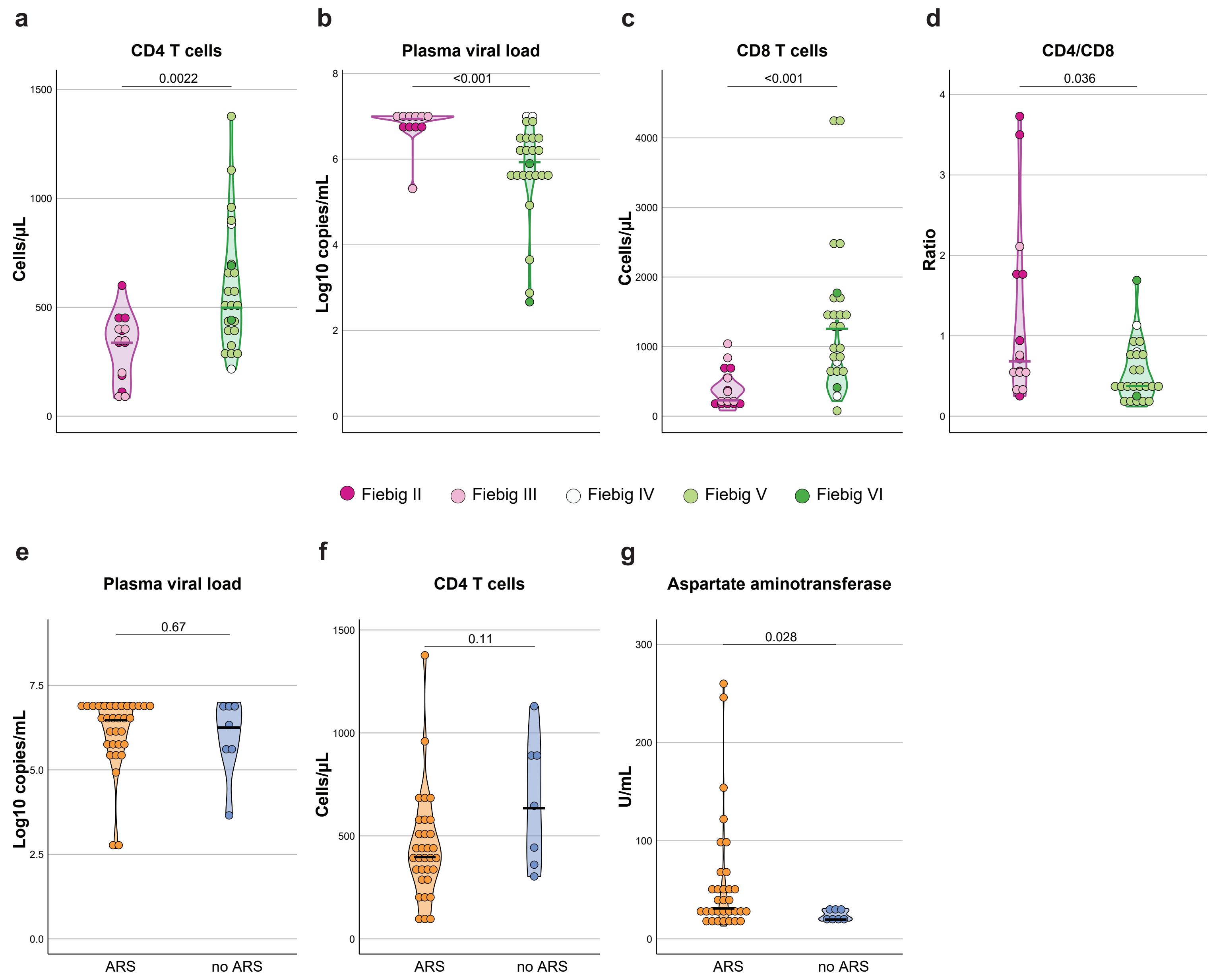

Supplementary figure 1
a, b, c, d, Violin plots displaying absolute CD4 T cell count **(a)**, HIV plasma viral load **(b)**, absolute CD8 T cell count **(c)** and CD4/CD8 ratio **(d)** at diagnosis (T0) in HIV+ participants diagnosed and treated in Fiebig stages II-III (purple) and Fiebig stages IV-VI (green). Dots are coloured according to Fiebig stages. P-values are determined by Mann-Whitney tests. **e, f, g,** Violin plots displaying HIV plasma viral load **(e)**, absolute CD4 T cells count **(f)**, and AST values **(g)** at diagnosis (T0) in HIV+ participants presenting with (orange) and without (blue) ARS. P-values are determined by Mann-Whitney tests. Abbreviations: AST= aspartate aminotransferase, ARS= acute retroviral syndrome

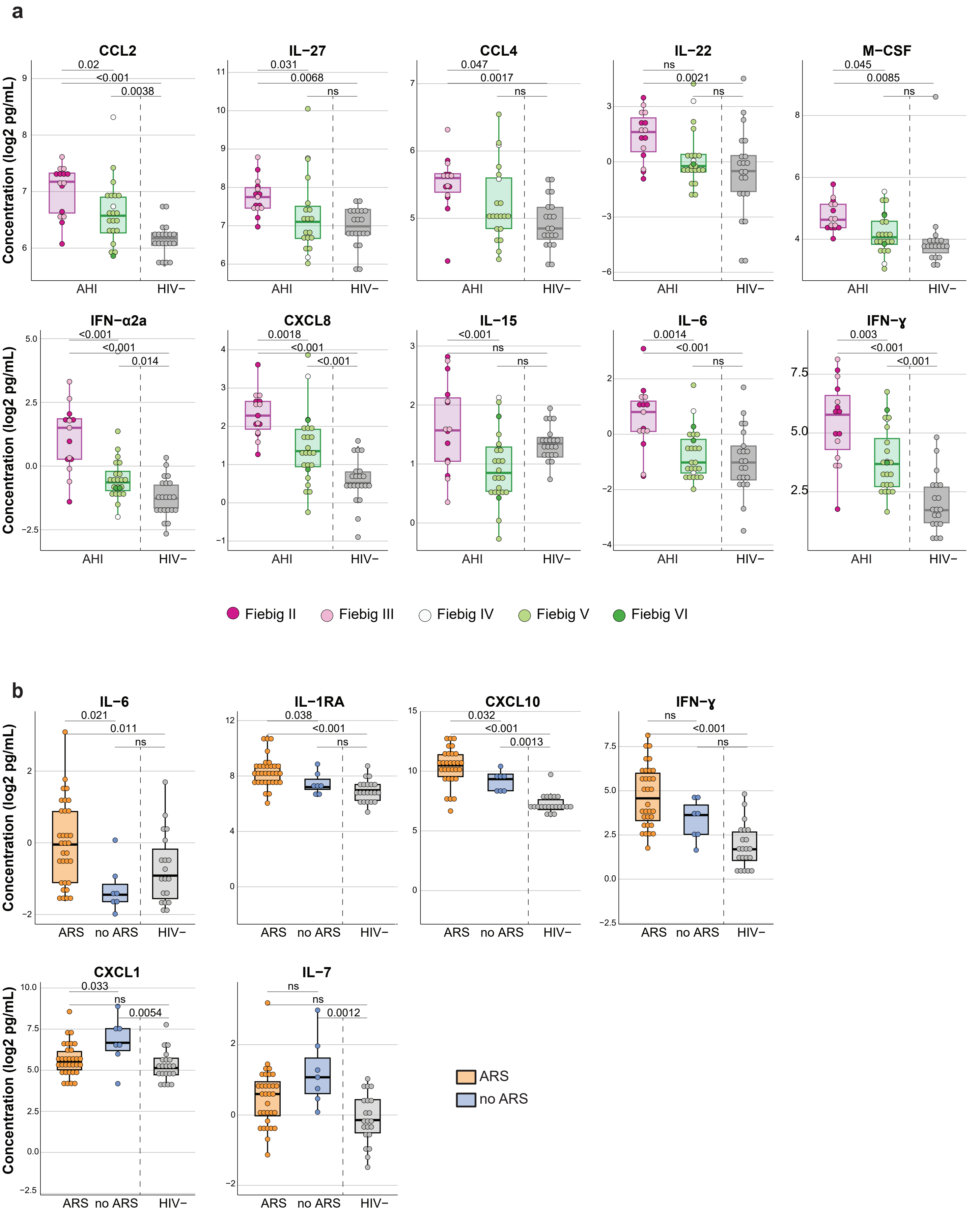

Supplementary figure 2

a, Box plots displaying plasma concentrations of CCL2, IL-27, CCL4, IL-22, M-CSF, IFN-α2a, CXCL8, IL-15, IL-6, and IFN-γ at diagnosis (T0) in HIV+ participants diagnosed and treated in Fiebig stages II-III (purple) and Fiebig stages IV-VI (green) and in HIV- participants (grey). Dots are colored according to Fiebig stages. **b,** Box plots displaying plasma concentrations of IL-6, IL-1RA, CXCL10, IFN-γ , CXCL1, and IL-7 at diagnosis (T0) in HIV+ participants presenting with (orange) and without (blue) ARS and in HIV- participants (grey). P-values were calculated using a linear model with batch as a fixed factor. All concentrations were batch-corrected and log2 transformed. Abbreviations: AHI= acute HIV infection, ARS= acute retroviral syndrome

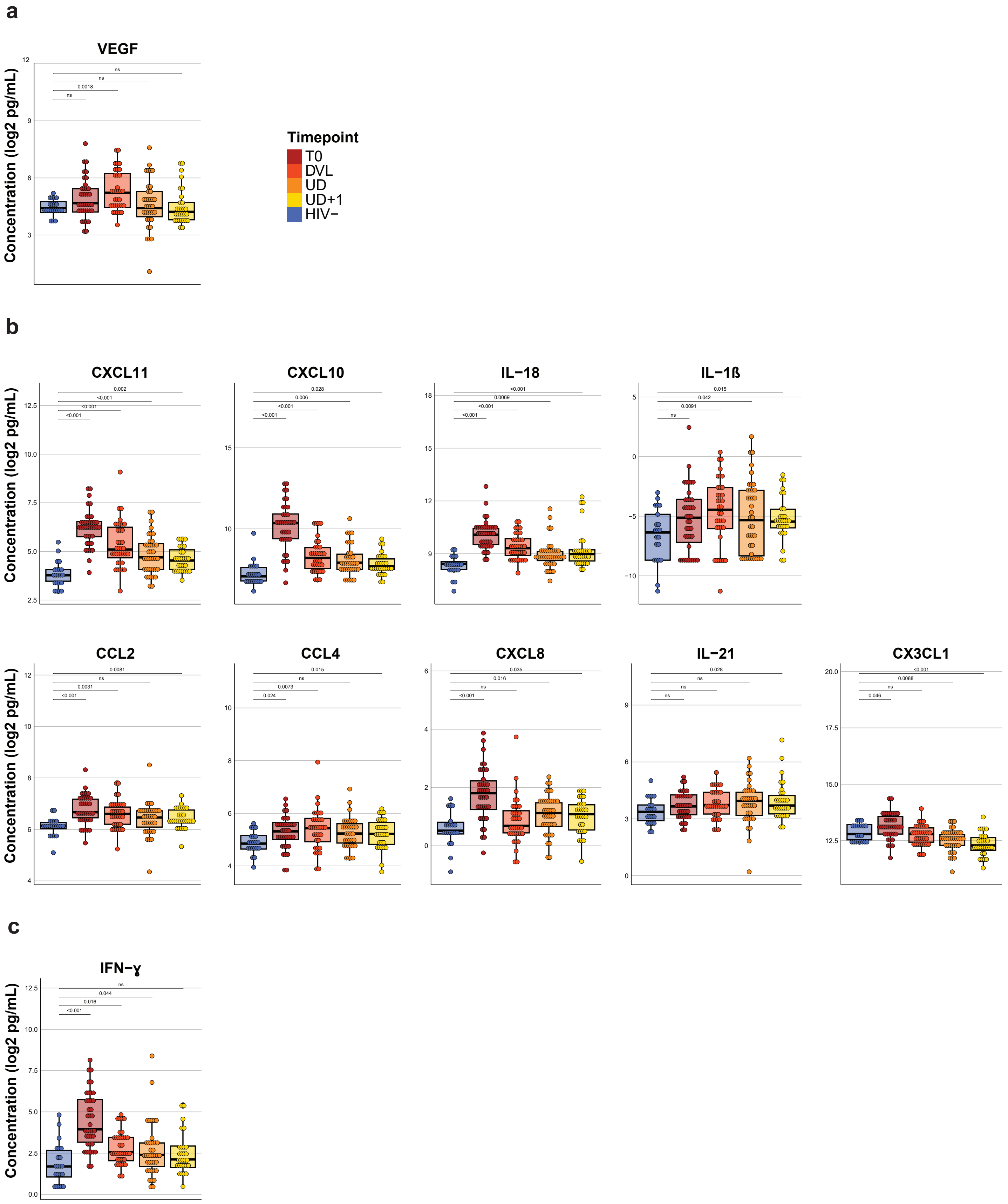

Supplementary figure 3
a, b ,c, Box plots displaying batch-corrected log2 transformed plasma concentrations of VEGF **(a)**, CXCL11, CXCL10, IL-18, IL-1β, CCL2, CCL4, CXCL8, IL-21, CX3CL1 **(b)** and IFN-γ **(c)** in HIV- (blue) and HIV+ participants at four timepoints: T0 (burgundy), DVL (dark orange), UD (light orange) and UD+1(yellow). P-values were calculated using a linear mixed regression model with timepoint and batch as fixed predictors.

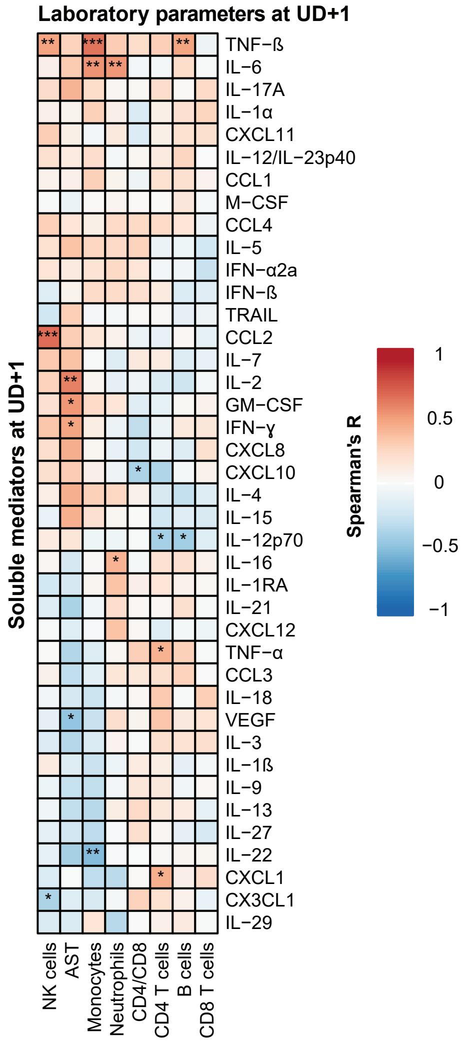

Supplementary figure 4: Correlation matrix of batch-corrected log2 transformed plasma concentrations of all measured soluble mediators during suppressed viremia (UD+1) and laboratory parameters (UD+1). Correlation coefficients and p-values were calculated with Spearman's rank test for pairwise complete observations. Positive and negative correlations are depicted in red and blue, respectively. Significant correlations are indicated by *, p<0·05; **, p<0·01; ***, p<0·001. Abbreviations: AST= aspartate aminotransferase

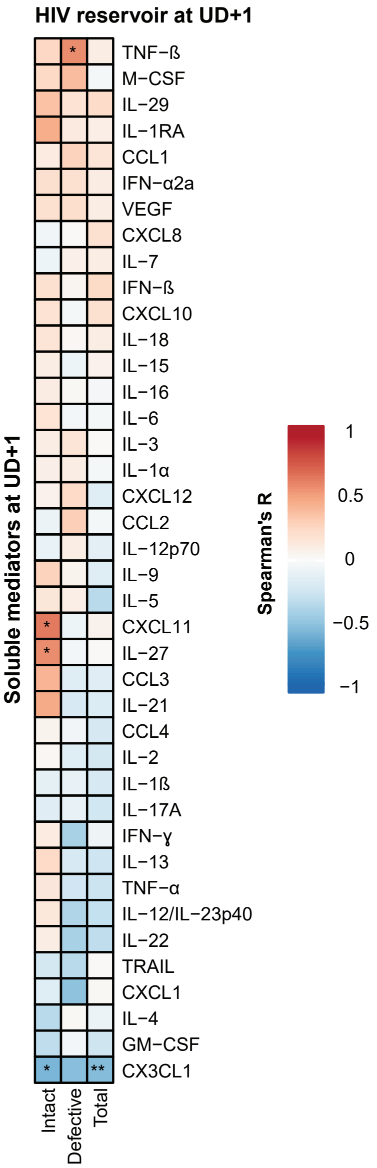

Supplementary figure 5: Correlation matrix of batch-corrected log2 transformed plasma concentrations of all measured soluble mediators during suppressed viremia (UD+1) and intact (n=14), defective (n=14) and total (n=29) HIV DNA (UD+1). Correlation coefficients and p-values were calculated with Spearman's rank test for pairwise complete observations. Positive and negative correlations are depicted in red and blue, respectively. Significant correlations are indicated by *, p<0·05; **, p<0·01; ***, p<0·001.
